# Identification and characterization a novel polar tube protein (NbPTP6) from the microsporidian *Nosema bombycis*

**DOI:** 10.1186/s13071-020-04348-z

**Published:** 2020-09-15

**Authors:** Qing Lv, Lijun Wang, Youpeng Fan, Xianzhi Meng, Keke Liu, Bingqian Zhou, Jie Chen, Guoqing Pan, Mengxian Long, Zeyang Zhou

**Affiliations:** 1 State Key Laboratory of Silkworm Genome Biology, Southwest University, Chongqing, 400715 China; 2 Chongqing Key Laboratory of Microsporidia Infection and Control, Southwest University, Chongqing, 400715 China; 3 College of Life Sciences, Chongqing Normal University, Chongqing, 400047 China

**Keywords:** *Nosema bombycis*, Novel, Polar tube protein, Localization, Cell-binding ability

## Abstract

**Background:**

Microsporidians are opportunistic pathogens with a wide range of hosts, including invertebrates, vertebrates and even humans. Microsporidians possess a highly specialized invasion structure, the polar tube. When spores encounter an appropriate environmental stimulation, the polar tube rapidly everts out of the spore, forming a 50–500 µm hollow tube that serves as a conduit for sporoplasm passage into host cells. The polar tube is mainly composed of polar tube proteins (PTPs). So far, five major polar tube proteins have been isolated from microsporidians. *Nosema bombycis*, the first identified microsporidian, infects the economically important insect silkworm and causes heavy financial loss to the sericulture industry annually.

**Results:**

A novel polar tube protein of *N. bombycis* (NbPTP6) was identified. NbPTP6 was rich in histidine (H) and serine (S), which contained a signal peptide of 16 amino acids at the N-terminus. NbPTP6 also had 6 potential O-glycosylation sites and 1 potential N-glycosylation site. The sequence alignment analysis revealed that NbPTP6 was homologous with uncharacterized proteins from other microsporidians (*Encephalitozoon cuniculi*, *E. hellem* and *N. ceranae*). Additionally, the NbPTP6 gene was expressed in mature *N. bombycis* spores. Indirect immunofluorescence analysis (IFA) result showed that NbPTP6 is localized on the whole polar tube of the germinated spores. Moreover, IFA, enzyme-linked immunosorbent (ELISA) and fluorescence-activated cell sorting (FACS) assays results revealed that NbPTP6 had cell-binding ability.

**Conclusions:**

Based on our results, we have confirmed that NbPTP6 is a novel microsporidian polar tube protein. This protein could adhere with the host cell surface, so we speculated it might play an important role in the process of microsporidian infection.
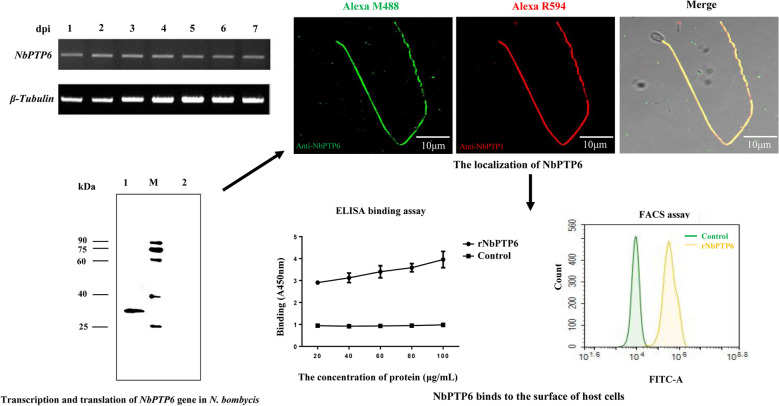

## Background

Microsporidians are obligate, intracellular, spore-forming parasites and currently considered as a kind of fungi [1–4]. Since the discovery of the first microsporidian *Nosema bombycis* from silkworms in the 19th century, there are some microsporidians that infect immunocompromised humans such as *Encephalitozoon hellem* (Eh), *E. cuniculi* (Ec) and *Enterocytozoon bieneusi* (Eb) [1, 5]; other microsporidians are insect pathogens such as *N. bombycis* (Nb), *N. ceranae* (Nc), and *Antonospora locustae* (Al) [6–8]. Although microsporidians differ in host range and specificity, they all utilize a similar infection mechanism. Under a certain external stimulation, the highly specialized polar tube is everted from the spore, forming a hollow tube that delivers the infectious sporoplasm into its host cell’s cytoplasm [1, 9–11]. However, due to the small size of the spores (1–10 µm), the small diameter of the polar tube (0.1–0.2 µm), and the rapidity of polar tube everting and sporoplasm passage (< 2 s), the study of the polar tube extrusion process is difficult [6–8, 12, 13].

The polar tube is composed of proteins, and presently, five kinds of polar tube proteins (PTP1-PTP5) have been identified from different microsporidia. PTP1, a proline-rich protein, contains many cysteine residues in its N-terminal and C-terminal domains. PTP1 is an O-mannoglycosylated protein that presumably binds to host sugar receptors to promote microsporidian infection [8, 11, 14, 15]. Interestingly, PTP2 is found at the same genomic locus with PTP1 [14]. Despite a high sequence divergence, various PTP2 proteins share common characteristics, such as the protein molecular weight, basic isoelectric point (pI) and high lysine content. The conservation cysteine residues of PTP2 are most likely involved in intra- and/or inter-protein disulfide bridges [15–18]. The third polar tube protein, PTP3, is identified from an *E. cuniculi* cDNA library. PTP1, PTP2 and PTP3 have been demonstrated to interact with each other by yeast two-hybrid assays [19, 20]. Two new PTPs (PTP4 and PTP5) have been identified from the polar tube by proteomics and antibody-based approaches [21, 22]. Recently, transferrin receptor 1 (TfR1) was identified as a potential host cell receptor of EhPTP4, and the microsporidian infection rate in TfR1-knockout mutant host cells was markedly reduced [23].

In this study, we identified a novel polar tube protein from *N. bombycis* named as NbPTP6. The specific polyclonal antibody of this protein labeled the whole polar tube of geminated spores. Furthermore, this polar tube protein could bind with the surface of host cells, suggesting it might play an important role in the process of *N. bombycis* infection.

## Methods

### Sequence analysis

NbPTP6 was screened from the proteomic data of germinated spores (Additional file 1: Figure S1; Additional file 2: Table S1). To further identify and functionally characterize NbPTP6, protein conserved domains were predicted by online tool (http://smart.emblheidelberg.de/index2.cgi). The signal peptide was predicted by SignalP (http://www.cbs.dtu.dk/services/SignalP/). Multiple sequence alignment was generated with ClustalX1.83 [24] and phylogenetic relationships were assessed with MEGA5 program [24]. The phosphorylation site was predicted using the SCANPROSITE tool (http://prosite.expasy.org/prosite.html). NETNGLYC (http://www.cbs.dtu.dk/services/NetNGlyc/) and NETOGLYC (http://www.cbs.dtu.dk/services/NetOGlyc/) were used to analyze N- and O-glycosylation sites, respectively. The secondary structure of the protein was predicted by using the Jpred 4 tool (http://www.compbio.dundee.ac.uk/jpred/).

### RNA extraction and semi-quantitative reverse transcription polymerase chain reaction (sqRT-PCR)

The sqRT-PCR was applied to detect the transcription level of the *NbPTP6* gene. Fifth-instar silkworms were infected with 1 × 10^4^/ml virulent *N. bombycis*. The midgut of infected and control silkworms were collected at 1–7 days post-infection. The total RNA was extracted and reverse-transcribed into cDNA using reverse transcription kit (Promega, Shanghai, China). PCR primers were designed by Primer Premier 5.0 (Table 1) according to the *NbPTP6* gene (GenBank: KB910042) and *β-tubulin* gene (GenBank: ABG54480.1). PCR conditions were as follows: 95 °C for 5 min, 30 cycles of 95 °C for 40 s, 55 °C for 40 s and 72 °C for 1 min.

**Table 1:** Oligonucleotide primers for this study

Gene name	Primer name	Primer sequence (5′–3′)	Fragment length (bp)
*NbPTP6* (for cloning)	NbPTP6_F	CGC**GGATCC**GAGCAGTTTAAACTTAAACA	693
	NbPTP6_R	CCG**CTCGAG**TTATTGATTCATAAAATTCA	
*NbPTP6* (for sqRT-PCR)	NbPTP6_F	ATGAAATTAATAATGATTA	741
	NbPTP6_R	TTATTGATTCATAAAATTC	
*β-tubulin*	β-tubulin_F	ATGAGAGAAATTATTCACTT	737
	β-tubulin_R	TTAATTTCCCATATAATCCC	

*Notes*: Restriction sites are indicated in bold

### Cloning, expression, purification of recombinant NbPTP6 and preparation of antiserum against NbPTP6

Primers for NbPTP6 cloning were designed without signal peptide, as shown in Table 1. The amplified DNA fragment without signal peptide of NbPTP6 (693 bp) was inserted into pMD19-T vector for sequencing and subcloned into pET32a (+) plasmid. The recombinant plasmid was transformed into *Escherichia coli* strain BL21 (DE3) for prokaryotic expression. Cells were cultured and induced at 37 °C with 0.2 M IPTG for 4 h, then the recombinant proteins were purified using a Ni-NTA affinity chromatography column (Qiagen, Beijing, China). Mice were immunized with rNbPTP6 protein mixed with Freund’s adjuvant (1:1; Sigma-Aldrich, St. Louis, USA). After four immunizations at intervals of 7 days, anti-NbPTP6 serum was obtained and stored at − 20 °C.

### Western blot analysis

The extraction method of total spore protein of mature spores was referred by Liu et al. [21]. Concisely, spores (1 × 10^9^ spores/ml) were disrupted 5 min for 3 times with 0.4 g acid-washed glass beads (0.2 g, 212–300 μm; 0.2 g, 425–600 μm; Sigma-Aldrich) in 500 μl buffer PBS (pH 7.4), then centrifuged at 12,000× *rpm* for 5 min at 4 °C. The supernatant was collected as the total spore protein [24]. For immunoblotting analysis, total spore protein was separated by 12% SDS-PAGE and transferred to a PVDF membrane (Roche, Shanghai, China). The membrane was incubated with mouse anti-NbPTP6 serum (diluted 1:500 in blocking solution) for 1 h at 37 °C. The anti-Trx-Tag mouse monoclonal antibody (1:1000 dilution; Sangon Biotech, Shanghai, China) was used as a negative control. Then the membrane was washed and incubated with anti-mouse lgG secondary antibody (1:8000 dilution; Sigma-Aldrich) for 1 h at 37 °C. The blot was developed with ECL western blot detection kit (Thermo Fisher Scientific, Shanghai, China).

### Immunofluorescence analysis (IFA)

Purified mature spores were added on poly-lysine coated slides and dried at room temperature for 30 min. Then, spores were germinated in 0.1 M KOH for 40 min at 30 °C, fixed in 4% formaldehyde at room temperature for 30 min, washed and blocked with 10% (v/v) non-specific goat serum together with 5% (w/v) bovine serum albumin (BSA) in PBST for 1 h at room temperature. Then, samples were simultaneously incubated with mouse anti-NbPTP6 (1:100 dilution) and rabbit anti-NbPTP1 (1:200 dilution) serum at room temperature for 1 h. Meanwhile, the anti-Trx-Tag mouse monoclonal antibody was used as a negative control. After washing three times, samples were incubated with Alexa Fluor® 594 conjugate goat anti-rabbit IgG (1:1000 dilution; Thermo Fisher Scientific) and Alexa Fluor® 488 conjugate goat anti-mouse IgG (1:1000 dilution; Thermo Fisher Scientific) for 1 h at room temperature. After washing with PBST, samples were stained with DAPI (1:1000 dilution; Thermo Fisher Scientific) for 20 min and examined under an Olympus FV1200 laser confocal microscope (Olympus, Tokyo, Japan).

To detect the binding of NbPTP6 by IFA, BmE cells were grown in 12-well plates and fixed with 4% paraformaldehyde in PBS for 30 min at room temperature, washed and blocked with 10% (v/v) non-specific goat serum together with 5% (w/v) bovine serum albumin (BSA) in PBST for 1 h at room temperature. After washing three times with PBST, rNbPTP6 protein was added to the well and incubated at 37 °C for 1 h. The pET-32a (+) non-load protein at the same concentration as rNbPTP6 was used as a negative control for these assays. After washing three times with PBST, samples were incubated with a commercial His-tag mouse monoclonal antibody (1:1000 dilution; Sigma-Aldrich) at room temperature for 1 h. After washing three times, the samples were incubated with Alexa Fluor® 488 conjugate goat anti-mouse IgG (1:1000 dilution; Thermo Fisher Scientific) for 1 h at room temperature. After washing with PBST, samples were stained with DAPI (1:1000 dilution; Thermo Fisher Scientific) for 20 min and examined under an Olympus FV1200 laser confocal microscope (Olympus).

### ELISA binding assay

Embryonic cells of *Bombyx mori* (BmE cells) were grown in 96-well plates and fixed with 4% paraformaldehyde in PBS for 30 min at room temperature. Non-specific binding sites were blocked by incubating with 3% BSA in TBST buffer. After washing three times with TBST, different concentration of the rNbPTP6 protein was added to each well and incubated at 37 °C for 1 h. The plates were washed three times with TBST, and fixed by incubating with methanol for 10 min. After washing with TBST three times, a commercial His-tag mouse monoclonal antibody (1:1000 dilution; Sigma-Aldrich) was added to cell plates and incubated at room temperature for 1 h, and the cells were washed three times with TBST. Then, anti-mouse lgG secondary antibody (1:8000 dilution; Sigma-Aldrich) was added to cell plates and incubated at room temperature for 1 h, and the cells were washed three times with TBST. The pET-32a (+) non-load protein was used as a negative control. O-diaminobenzene (Sigma-Aldrich), as a kind of reaction substrate, was added to the plate at 37 °C for 15 min, then OD_450_ was detected using a Tecan microplate reader (Tecan, Shanghai, China).

### Fluorescence-activated cell sorting FACS assays

BmE cells (1 × 10^7^) were washed three times with PBS buffer. rNbPTP6 protein (0.5 g/ml) was incubated with BmE cells at 37 °C for 1 h, washed three times, then blocked with 10% (v/v) non-specific goat serum together with 5% (w/v) BSA in PBST at 37 °C for 1 h. After washing three times, the commercial His-tag mouse monoclonal antibody (1:1000 dilution; Sigma-Aldrich) was incubated with cells for 1 h at 37 °C then washed with PBST three times. Cells were then incubated with Alexa Fluor® 488 conjugate goat anti-mouse IgG (1:1000 dilution; Thermo Fisher) at room temperature for 1 h. After washing three times, cell samples were examined using a flow cytometer (Beckman Coulter, Brea, USA). The pET-32a (+) non-load protein was used as a negative control.

## Results

### Sequence characteristics of NbPTP6

**Fig. 1:**
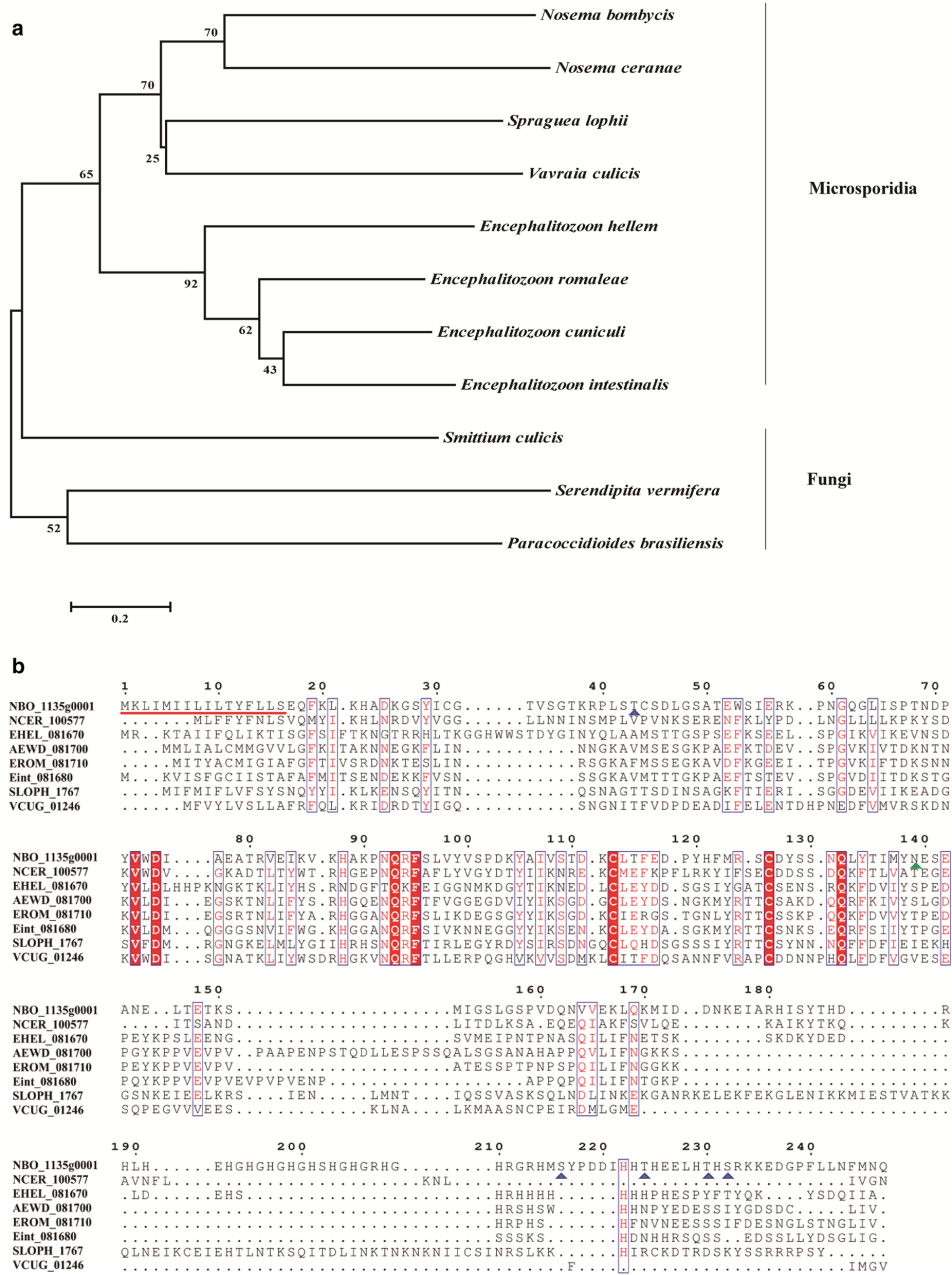
Alignment and phylogenetic tree of multiple NbPTP6 homologous sequences in various microsporidia, fungi and bacteria. **a** Phylogenetic tree of multiple NbPTP6 homologous sequences. Neighbour-joining tree was constructed using MEGA 5 software. The amino acid sequences were obtained from Uniprot: *E. cuniculi* (AEWD_081700); *E. hellem* (EHEL_081670); *N. ceranae* (NCER_100577); *V. culicis* (VCUG_01246); *Spr. lophii* (SLOPH_1767); *N. bombycis* (NBO_1135g0001); *E. romaleae* (EROM_081710); *E. intestinalis* (Eint_081680); *Smi. culicis* (AYI70_g11826); *P. brasiliensis* (PADG_06126); and *Ser. vermifera* (M408DRAFT_280892). **b** Alignment of NbPTP6 homologous sequences. The red shades highlight identical amino acids; the red underline indicates the predicted signal peptide of NbPTP6; the blue and green triangles represent O-glycosylation sites and N-glycosylation sites of NbPTP6, respectively

NbPTP6 gene is 741 bp in length and encodes a 247-amino acid (aa) protein, which has a calculated molecular weight of 28.3 kDa and a predicted pI of 7.26. The sequence is rich in histamine and has a signal peptide consisting of 16 amino acids at the N-terminal. Moreover, it has 6 potential O-glycosylation sites and 1 potential N-glycosylation site. The phosphorylation prediction result showed that NbPTP6 has 18 Ser phosphorylation sites, 8 Thr phosphorylation sites and 7 Tyr phosphorylation sites. The protein has a simple helix structure, which is similar to the structure of the known polar tube proteins. However, there is no specific known conserved domain in NbPTP6. The phylogenetic tree showed that the NbPTP6 protein is closely related to a certain unknown protein of *N. ceranae*. More interestingly, NbPTP6 and homologous proteins of other microsporidians were in the same clade (Fig. 1a). The following NbPTP6 homologous protein sequences were found: from *E. cuniculi* (AEWD_081700; 26% identity); *E. hellem* (EHEL_081670; 24% identity); *N. ceranae* (NCER_100577; 27% identity); *E. romaleae* (EROM_081710; 28% identity); *E. intestinalis* (Eint_081680; 37% identity); *V. culicis* (VCUG_01246; 33% identity); and *S*. *lophii* (SLOPH_1767; 26% identity) (Fig. 1b).

### Transcription and translation of *NbPTP6*

**Fig. 2:**
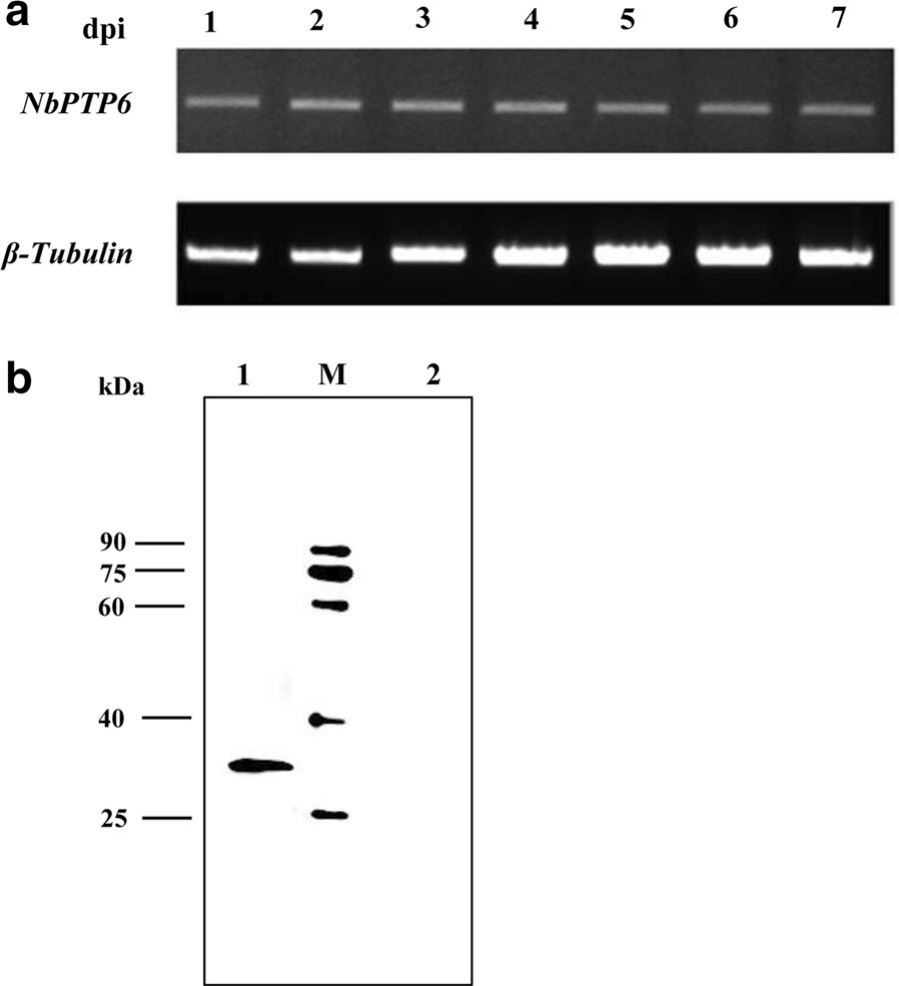
Transcription and translation of *NbPTP6* gene in *N. bombycis*. **a** The transcription pattern of *NbPTP6* was analyzed by semi-quantitative RT-PCR using the midgut cDNA of *N. bombycis*-infected silkworms at 1–7 dpi. The *β-tubulin* transcription of *N. bombycis* was used as the control. **b** Western blot analysis of NbPTP6 expressed in total spore protein of *N. bombycis*. Lane 1: immunoblotting of mouse anti-NbPTP6 serum in total spore protein; Lane 2: immunoblotting of mouse anti-Trx-Tag serum in total spore protein; Lane M: protein molecular marker (Fermentas, Shanghai, China)

To investigate the transcription level of *NbPTP6*, total mRNA from the midgut of infected silkworms at 1–7 dpi was extracted respectively. The sqRT-PCR results shown in Fig. 2 indicate that *NbPTP6* was transcribed in *N. bombyci*s-infected silkworms from 1 to 7 dpi (Fig. 2a). Western blot analysis indicated that a unique band (30 kDa) was detected in the total spore protein of *N. bombycis*, which is consistent with the theoretical molecular weight of NbPTP6 (Fig. 2b).

### Immunolocalization of NbPTP6

**Fig. 3:**
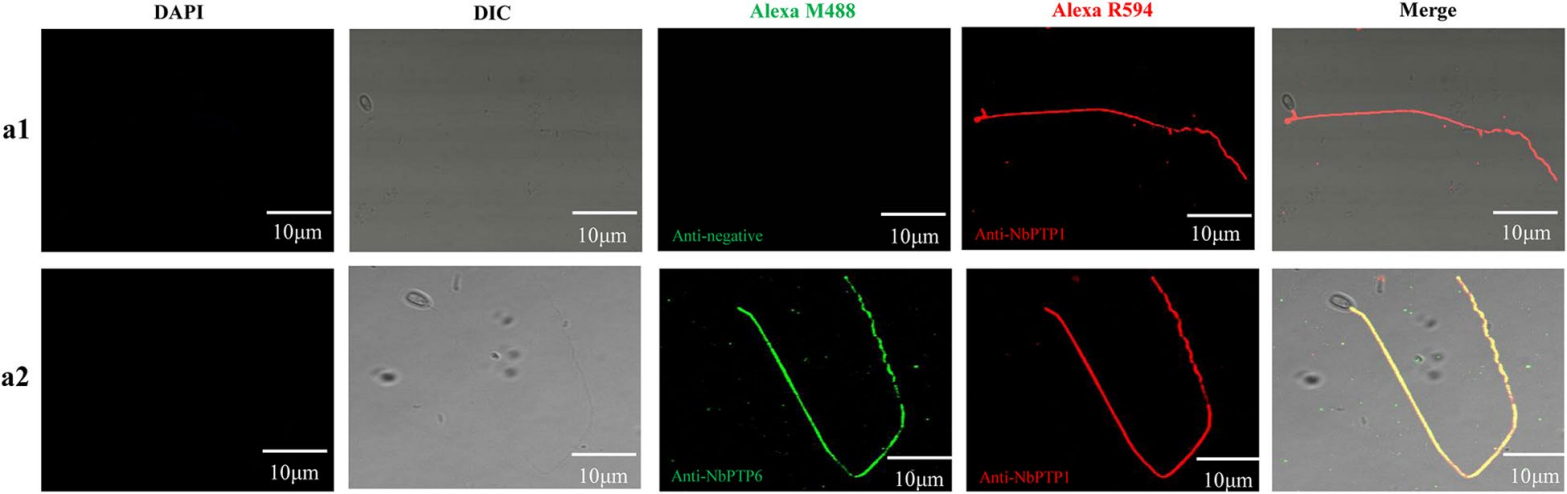
Immunofluorescence assay of NbPTP6 localization in germinated spores. **a1** The extruded polar tube was treated with mouse anti-Trx-Tag serum and rabbit anti-NbPTP1 serum. NbPTP1 was detected (red), and immunofluorescent signal of Trx-Tag could not be detected in polar tube. **a2** The extruded polar tube was treated with mouse anti-NbPTP6 serum and rabbit anti-NbPTP1 serum. NbPTP1 was detected (red), and NbPTP6(green) had co-localization with NbPTP1. The nuclei were labeled with DAPI. *Scale-bars*: 10 μm

We used germinated spores to study the localization of NbPTP6 in *N. bombyci*s. The IFA result revealed that anti-NbPTP6 serum labeled green fluorescence of Alexa-488 dye was localized on the whole polar tube, and anti-NbPTP1 serum labeled red fluorescence of Alexa-594 dye was also localized on the whole polar tube. Interestingly, the signal of anti-NbPTP6 serum and anti-NbPTP1 serum could completely merge together (Fig. 3b). The nuclei of *N. bombycis* spores were labelled for blue fluorescence with DAPI. After germination, the spore nuclei were ejected out of spores, so there was no blue signal in the empty spore shells. For the negative control group, no green fluorescence was detected by native mouse serum (Fig. 3a). In addition, by IEM, gold particles-labeled NbPTP6 antibody were distributed mainly in the polar tube region of spores (Additional file 3: Figure S2a1); however, no gold particles-labeled Trx-tag antibody was detected in the negative-control (Additional file 3: Figure S2a2). Therefore, these data confirmed that NbPTP6 is a novel polar tube protein of *N. bombycis*.

### Cell binding ability analysis of NbPTP6

**Fig. 4:**
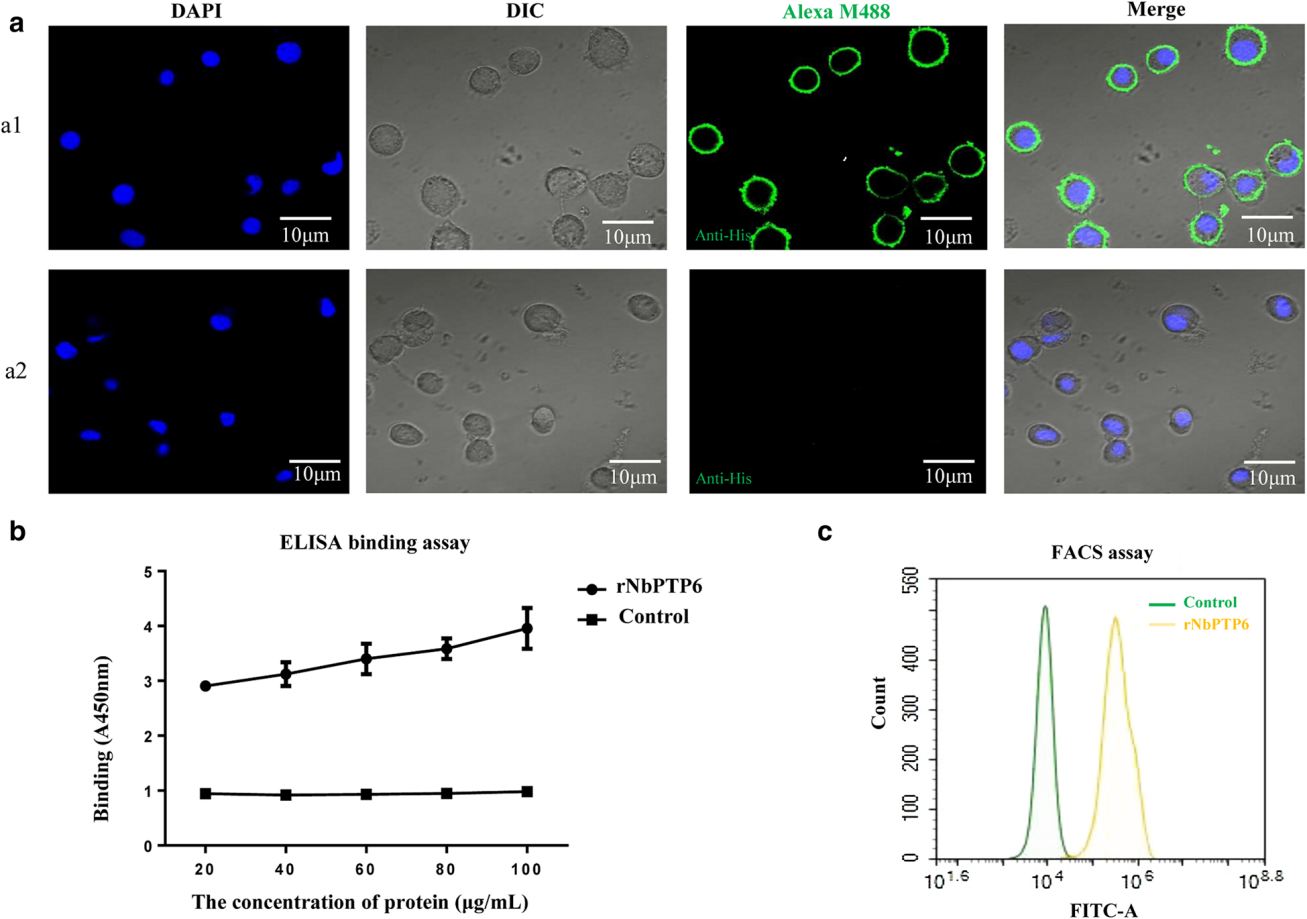
NbPTP6 binds to the surface of host cells. **a** IFA of NbPTP6 binding to BmE cells. **a1** rNbPTP6 proteins were incubated with BmE cells grown on glass coverslips and treated with mouse anti-His serum. And rNbPTP6 proteins were detected (green) around the cells. **a2** The pET-32a (+) non-load protein at the same concentration as rNbPTP6 was used as a negative control for these assays. The immunofluorescent signal of pET-32a (+) non-load proteins could not be detected. DAPI was used to stain the nuclei. *Scale-bars*: 10 μm. **b** ELISA detection of NbPTP6 binding to BmE cells. rNbPTP6 proteins with different concentrations were incubated with BmE cells in 96-well plates and binding was detected *via* ELISA. The pET-32a (+) non-load protein at the same concentration as rNbPTP6 was used as a negative control for these assays. **c** FACS analysis of NbPTP6 binding with BmE cells. Yellow curve represents the binding cell fluorescence intensity of rNbPTP6 protein, and green curve represents the binding cell fluorescence intensity of control protein

In order to explore the role of NbPTP6 in *N. bombycis* infection, we conducted a preliminary study on its host cell binding ability. First, IFA was used to test the hypothesis of the cell binding ability of NbPTP6. The purified rNbPTP6 protein was incubated with BmE cells. The green fluorescence from Alexa-488 dye labeled the commercial His-tag antibody was shown around the BmE cell, revealing that NbPTP6 could bind with the surface of BmE cells (Fig. 4a1). But in the control group, there was no green fluorescence (Fig. 4a2). Then, we further employed an ELISA to verify the cell-binding ability of NbPTP6, and the result showed that with the increase of protein concentration, the cell-binding ability of NbPTP6 became stronger, suggesting that NbPTP6 most likely interacts with a protein (or other binding partner) on the host cell membrane (Fig. 4b). Finally, we used FACS assays to further verify this hypothesis and found that the fluorescence intensity of the rNbPTP6 protein-bound cells was distinctly higher than the control group cells (Fig. 4c).

## Discussion

Microsporidians possess a unique invasion apparatus, the polar tube. Under the appropriate environmental stimulation, the polar tube can evert rapidly out of the microsporidian spore, form a hollow tube and serve as a conduit for the passage of sporoplasm and nuclear material into a new host cell [6, 7, 25]. Within the spore, the polar tube is filled with unpolymerized polar tube proteins. After germination, the length of the everted polar tubes is approximately 2–3 times longer than that of the coiled tubes inside spores, so it has been hypothesized that unpolymerized polar tube proteins are incorporated at the growing tip of the polar tube during everting or that the tube unfolds during eversion [1, 2, 25]. In this study, NbPTP6 was localized on the whole polar tube, which was the same as the localization of NbPTP1, NbPTP2 and NbPTP3. Based on these results, we determined that PTP6 is a novel polar tube protein of *N. bombycis.*

NbPTP6 had six potential O-glycosylation sites and one potential N-glycosylation site, which was similar to the NbPTP1 protein. Previously, O-glycosylation of EhPTP1 was found to affect the ability of *E. hellem* to infect host cells [26–28]. Moreover, NbPTP1 could bind with ConA, proving the existence of glycosylation modification [1, 23, 28, 29]. It was speculated that it might play an important role in protecting the polar tube from the host intestinal digestion, or in interacting with the mannose receptor on the host cell membrane to promote infection and adhesion of the polar tube to the host cell membrane [1, 23, 29].

Previous studies found that the polar tube is surrounded by the host cell membrane at the invasion site. And polar tube does not pierce or break the host plasma membrane, instead it is pushing the host cell plasma membrane into the host cell creating a microenvironment into which the microsporidian sporoplasm is extruded from the end of the polar tube [30–33]. Therefore, it was suggested there was an interaction between the polar tube and host cell membrane. In previous research, PTP4 from *E. hellem* had the ability to adhere with cells and TfR1 was identified as a potential host cell interacting receptor for EhPTP4. The infection rate of *E. hellem* was significantly reduced when TfR1 recombinant protein or anti-TfR1 antibody was added into the cell culture [23]. These results indicated that PTP4 is an important protein of the polar tube involved in the host cell infection mechanism of microsporidians. In our study, IFA, ELISA and FACS results confirmed that NbPTP6 could bind with host BmE cells, which was similar with EhPTP4. So we speculated that there were interaction receptors of NbPTP6 on the surface of host cells to facilitate microsporidian infection. We plan to screen and identify the potential host cell receptor of NbPTP6.

## Conclusions

In summary, we have identified a novel polar tube protein identified in the microsporidium *N. bombycis*. NbPTP6 was localized on the polar tube, and it has been shown to bind with the surface of host cells. It was helpful to understand the composition of polar tube and the infection mechanism of microsporidia.

## Supplementary information

**Additional file 1: Figure S1.** SDS-PAGE analysis the protein of germinated spores.

**Additional file 2: Table S1.** The top 20 proteins identified in the proteomic data of germinated spores.

**Additional file 3: Figure S2.** IEM analysis of NbPTP6 localization in microsporidian spores. a1 Mature *N. bombycis* spore, with gold particles labeled NbPTP6 antibody localized mainly to the polar tube region. The inset shows an enlarged section of the image. a2 Negative control. Arrowheads mark colloidal gold particles. *Scale-bar*: 500 nm. *Abbreviations*: En, endospore; Ex, exospore; PT, polar tube.

## Data Availability

Data supporting the conclusions of this article are included within the article and its additional files. The raw data are available from the corresponding author upon reasonable request.

## Abbreviations

PTPs: polar tube proteins
IFA: indirect immunofluorescence analysis
ELISA: enzyme-linked immunosorbent
FACS: fluorescence-activated cell sorting
sqRT-PCR: semi-quantitative reverse transcription polymerase chain reaction
BmE cells: embryonic cells of *Bombyx mori*
TfR1: transferrin receptor 1
IEM: immunoeletron microscopy
