# Supplementary figures and images for: Identification and characterization a novel polar tube protein (NbPTP6) from the microsporidian Nosema bombycis
Source: Parasit Vectors. 2020 Sep 15;13:475. doi: 10.1186/s13071-020-04348-z (PMC7493173; doi:10.1186/s13071-020-04348-z)

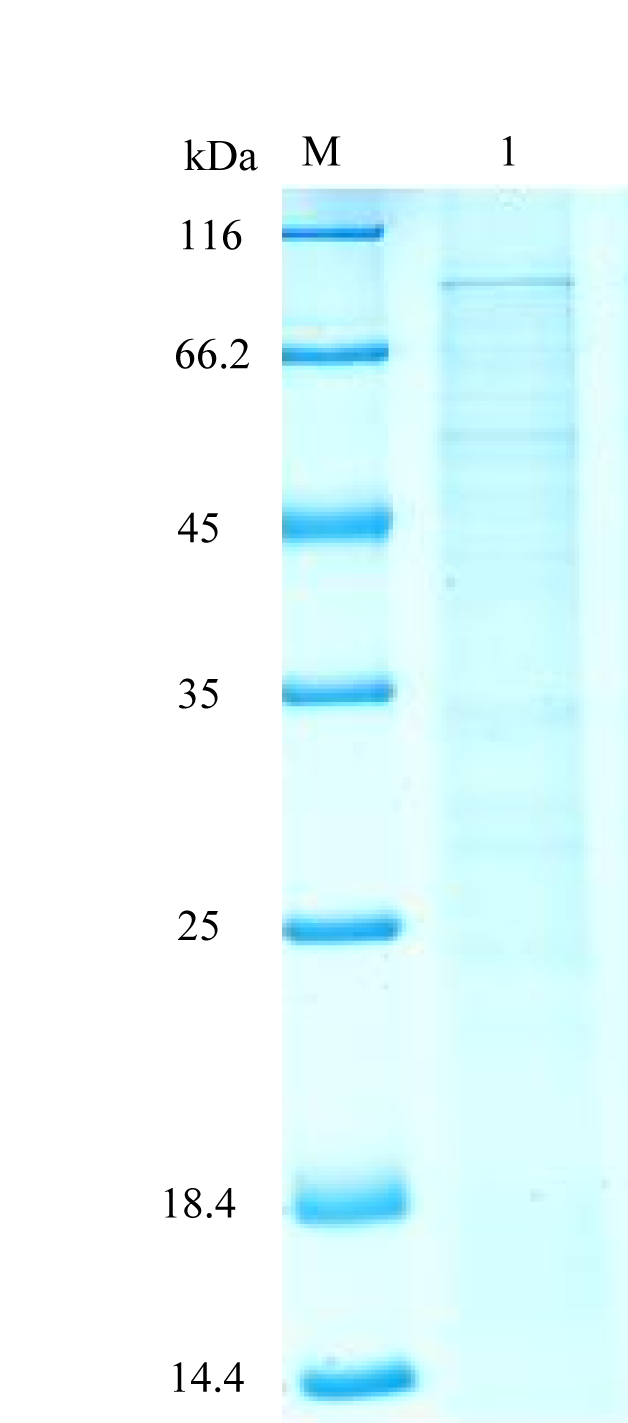

Supplement: Supplementary file 1 — Additional file 1: Figure S1. SDS-PAGE analysis the protein of germinated spores. [file 13071_2020_4348_MOESM1_ESM.tif]

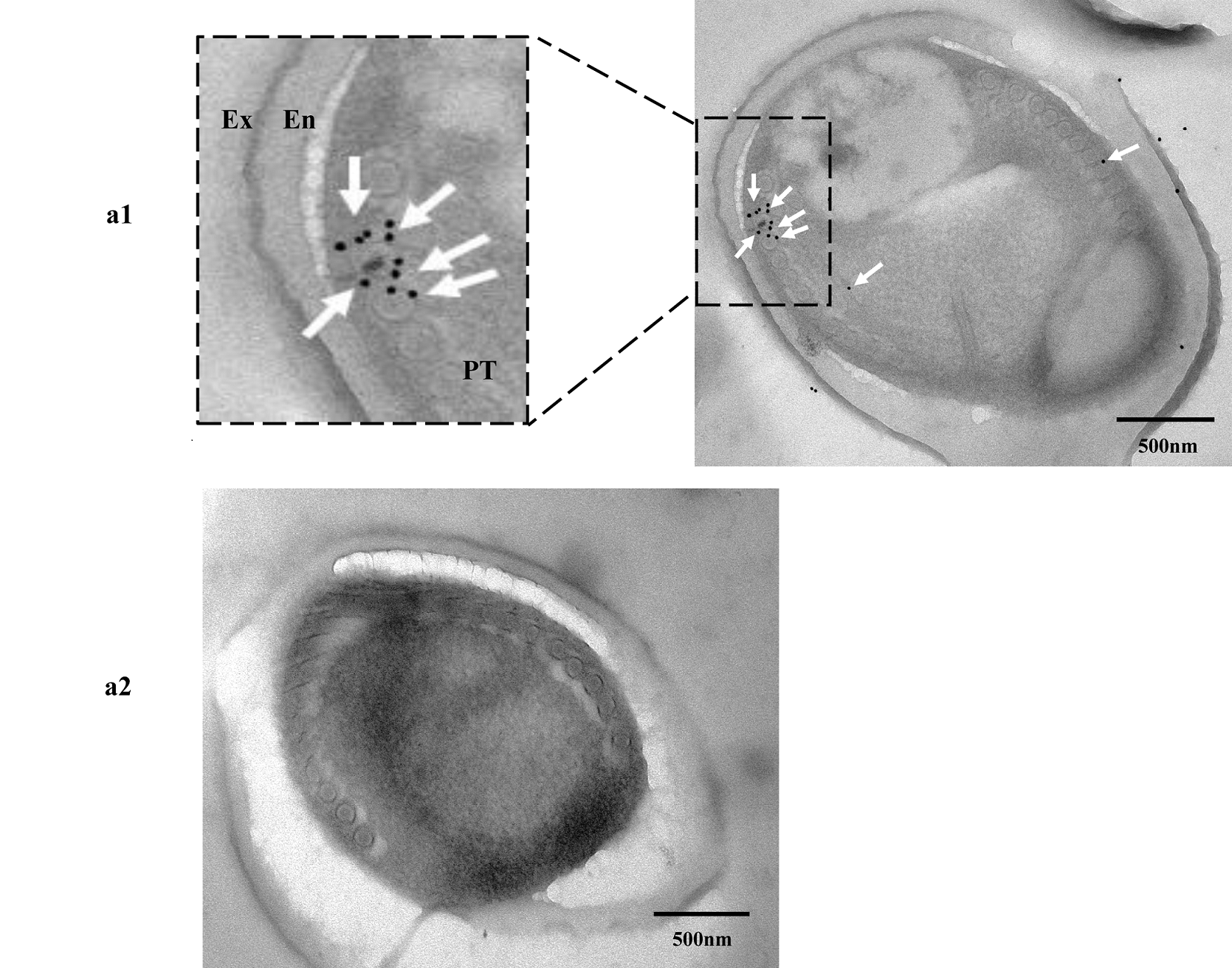

Supplement: Supplementary file 3 — Additional file 3: Figure S2. IEM analysis of NbPTP6 localization in microsporidian spores. a1 Mature N. bombycis spore, with gold particles labeled NbPTP6 antibody localized mainly to the polar tube region. The inset shows an enlarged section of the image. a2 Negative control. Arrowheads mark colloidal gold particles. Scale-bar: 500 nm. Abbreviations: En, endospore; Ex, exospore; PT, polar tube. [file 13071_2020_4348_MOESM3_ESM.tif]
