# Supplementary material for: Identification and characterization a novel polar tube protein (NbPTP6) from the microsporidian Nosema bombycis
Source: Parasit Vectors. 2020 Sep 15;13:475. doi: 10.1186/s13071-020-04348-z (PMC7493173; doi:10.1186/s13071-020-04348-z)
Supplement: Supplementary file 2 — Additional file 2: Table S1. The top 20 proteins identified in the proteomic data of germinated spores. [file 13071_2020_4348_MOESM2_ESM.docx]

| Protein IDs | Protein Name | Unique peptides | Sequence coverage [%] | iBAQ |
| --- | --- | --- | --- | --- |
| R0MJE4 | Uncharacterized protein | 7 | 34.6 | 4.02E+09 |
| R0KT65 | Uncharacterized protein | 19 | 39.4 | 3.87E+09 |
| R0KS75 | Putative membrane protein ycf1 | 42 | 71.7 | 3.47E+09 |
| R0MN31 | Elongation factor 1-alpha | 10 | 74.6 | 3.37E+09 |
| R0M670 | Uncharacterized protein | 4 | 55.2 | 3.21E+09 |
| R0MIQ9 | Histone H3 | 6 | 28.6 | 3.19E+09 |
| R0MIU3 | Eukaryotic translation initiation factor 2C | 41 | 65.8 | 2.75E+09 |
| R0KY97 | Polar tube protein 2 | 22 | 69.7 | 2.52E+09 |
| R0MIT2 | Heat shock 70 kDa protein 6 | 50 | 60.7 | 2.19E+09 |
| B3STP6 | Spore wall protein 12 | 18 | 78.9 | 1.85E+09 |
| R0M8Z2 | Actin | 20 | 74.1 | 1.8E+09 |
| R0KUF7 | Uncharacterized protein | 10 | 33 | 1.72E+09 |
| R0MC17 | Uncharacterized protein | 8 | 56.2 | 1.53E+09 |
| R0KVJ2 | Uncharacterized protein | 8 | 37.7 | 1.03E+09 |
| R0KPG7 | Uncharacterized protein | 17 | 54.8 | 9.60E+08 |
| R0MBR8 | Uncharacterized protein | 15 | 52.9 | 9.16E+08 |
| R0M6E4 | Aldose reductase | 10 | 62.4 | 8.68E+08 |
| R0MKI4 | Uncharacterized protein | 4 | 60.8 | 8.55E+08 |
| R0KTP7 | Uncharacterized protein | 11 | 58.8 | 8.23E+08 |
| R0MQM8 | Polar tube protein 1 | 9 | 63.6 | 8.11E+08 |

**Additional file 2: Table S1.** **The top 20 proteins identified from the proteomic data of the germinated spores.**

After spore germination, the spore protein sample was obtained by dissolving with DTT, urea, SDS and other solvents. The protein samples were identified by shotgun analysis (Shanghai Applied protein Technology Co. Ltd). The MS data were analyzed by MaxQuant software version 1.5.3.17 (Max Planck Institute of Biochemistry in Martinsried, Germany).
